# Risk rating in the tea planting industry: The employees’ opinion

**DOI:** 10.4103/0019-5278.75698

**Published:** 2010

**Authors:** Bobby Joseph, Christie Minj

**Affiliations:** Department of Community Health, St. John’s Medical College, Bangalore, India

**Keywords:** Hazards, injury, risk rating, tea planting industry, workers

## Abstract

**Background:**

Workers in the tea planting industry are exposed to a variety of occupational health and safety hazards. Whether the workers perceive the risks involved and to what degree is an interesting point in question.

**Aims:**

To identify occupational health and safety risks involved in the tea planting sector and to rate these risks from the workers’ perspective.

**Settings and Design:**

Permanent workers from four estates belonging to one tea planting company in southern India were enlisted in this descriptive study

**Materials and Methods:**

The sample was randomly and then proportionately selected to give a total number equal to the calculated sample size of 341. Data were collected by reviewing medical records, conducting focus group discussions with field officers and supervisors, worker interviews and key informant interviews with the management in these four estates. Proportions were used to describe occurrence and distribution of work-related injuries. The risks as perceived by the workers were rated on their severity and frequency, using a Risk Rating Matrix.

**Results and Conclusion:**

The incidence of injuries was greater among male workers, those working both in the field and factory and those handling multiple tasks. The most common morbidities suffered were “small cuts and abrasions” in about 53%of the workers. Backache and insect bites were assigned the highest risk rating scores. Continued monitoring of the risk assessment by the workers could help in a planned reduction of commonly occurring injuries by agreeing on a specified risk limit.

## INTRODUCTION

The tea planting industry in southern India accounts for 66,050 planters and employs a population of about 2.46 lakh as labor.[1] History reveals that most of the plantation areas were “distinctly unhealthy”[2] with primary health care issues such as communicable diseases and mother and child survival occupying the minds of the early planters. Over the years, planting companies have started focusing on occupation health and safety issues.

Workers in the tea planting industry are exposed to a variety of occupational health and safety hazards. In India, legislation to take care of occupational safety in tea plantations is also found wanting. Unlike the Factories Act,[3] the Plantations Labour Act[4] does not provide any guidance for occupational safety in the plantations.

This study was conducted with the objectives of identifying occupational health and safety risks involved in this sector and rating these risks from the workers’ perspective.

## MATERIALS AND METHODS

This was a descriptive study conducted in four selected estates belonging to one tea planting company that employs a total 3028 permanent workers in the fields and factories. A checklist of hazards and accidents likely to occur at the work place was derived after reviewing available literature, the medical records of the estate workers, the Workman’s Compensation register and by conducting focus group discussions and key informant interviews with the field officers, supervisory staff and managers of the estates and factories. The list of hazards identified was incorporated into a schedule of questions which were administered to the workers. Workers to be interviewed were selected by stratified random sampling using a list of employees for each estate – the strata considered being gender alone. Assuming that 50% of the workers had suffered an injury (at 95% confidence interval and a relative deviate of 10%), the sample size was estimated to be 341 for this finite number of workers.

After obtaining a checklist of hazards, the workers were asked to provide a *hazard rating* (based on severity of outcome) and *exposure rating* (based on frequency of exposure). Hazards were categorized by the perceived severity of the outcome (severity rating) into the following groups: 1 = first aid case; 2 = minor injury; 3 = major injury; 4 = fatality and 5 = multiple fatalities. The exposure rating was categorized by the perceived frequency of its occurrence (frequency rating) into the following groups: 1 = less than once a year; 2 = once a month; 3 = once a week; 4 = 2–3 times a week and 5 = daily. The Risk Rating Matrix was applied to each of the commonly occurring hazards or injuries in order to identify the injuries which would obtain the highest risk rating scores. The risks were then ranked in order of importance based on a commonly used method of risk rating Table 1.

**Table 1:** Risk rating interpretation based on perception of severity and frequency of injury

		Frequency				
		1	2	3	4	5
	1	Very Low	Very Low	Low	Low	Significant
**Severity**	2	Very low	Low	Significant	Significant	Significant
	3	Low	Low	Significant	High	High
	4	Low	Significant	Significant	High	High
	5	Low	Significant	High	High	High

Additionally, secondary data regarding the types of injuries, their frequency and management were obtained from various records and registers maintained by the estates. Proportions (percentages) were used to describe the occurrence and distribution of work-related injuries.

## RESULTS

Of the 341 workers surveyed, 299 (87.7%) were employed in the field, 13 (3.8%) were factory-based and 29 (8.5%) worked either in the field or in the factory depending on the need. The work in the field includes tasks such as plucking, weeding, pruning of tea bushes, manuring, spraying of chemicals by hand or using a powered equipment, and shade lopping (pruning branches of trees). The work in the factory includes operating machinery such as those for crushing, tearing and curling (CTC), winnowing, sorting and packaging of tea leaves and electrical works.

A gender-wise comparison of work-related injuries among the workers surveyed [Table 2] revealed that the incidence of injuries was far greater among males compared to females.

**Table 2:** Gender characteristics of the work-related injuries among surveyed workers

	Males	Females	Total
No. of workers surveyed	135	206	341
No. of man-days worked	41,175	62,830	104,005
No. of workers reporting injuries	35	41	76
Total no. of episodes	67	56	123
Incidence of workplace injuries in the past year	496.3/1000 workers	271.6/1000 workers	360.7/1000 workers
Injury rate	0.16/100 man-days	0.09/100 mandays	0.12/100 mandays

Among the 76 workers who reported work-related injuries in the past year, the most common morbidities suffered were “small cuts and abrasions” in 40 (52.6%) of the workers, “major cuts, abrasions and contusions” in 16 (21.1%), “foreign body in the eye” and ”sprains” in 5 (6.6%) each and insect bite in 4 (5.3%). Of these, 70 (92.1%) were considered minor – requiring only first aid at the work spot or minor interventions at the hospital (1 and 2 in the severity rating); the remaining 6 (7.9%) needed major intervention/referral at a higher center (3 in the severity rating).

None of those working only in the factory suffered from injuries. There was a significantly higher proportion of injuries among those working both in the field and factory as compared to those who worked only in the fields – 14 of 29 (48.3%) versus 62 of 299 (20.7%), respectively. Multi-tasking, defined as handling multiple tasks whether in the field or factory, resulted in a significantly higher incidence of injuries – 69 of 277 (24.9%) who “multi-tasked“ versus 7 of 64 (10.9%) who did not.

The review of medical records showed that during the past year there had been a total of 4203 hospital visits (mean 12.33, SD 10.82) among all the workers interviewed. Of these, 1651 (mean 12.23, median 9, mode 2, SD 11.85, range 60) had been among male workers and 2552 (mean 12.38, median 10.5, mode 2, SD 10.12, range 55) had been among female workers. The medical records also showed that 52 workers (30 males and 22 females) had visited a hospital/health facility at least once due to work-related accidents/injuries. The total number of hospital visits due to work-related injuries was 59 (30 male and 29 females). This accounted for 1.8% of all hospital visits among male workers and 1.1% of all hospital visits among female workers.

The interviewed workers did not identify any issues to be “significant” or “high” in terms of the risk rating using the criteria for severity and frequency mentioned earlier. The categorization of risks is depicted in Table 3.

**Table 3:** Categorization of injuries in the Risk Rating Matrix as perceived by workers

		Frequency				
		1	2	3	4	5
Severity	1	Sprains/dislocations, cuts/bruises, eye injury, solar radiation, fire/burns, chemical injuries	Heat, noise		Backache, insect bite	
	2	Amputation, fractures, dermatitis, electrical injuries, lightning, snake bites				
	3	Animal attack				
	4					
	5					

## DISCUSSION

It is estimated that 17 million occupational non-fatal injuries (17% of the world) occur each year in India.[5] There are no data pertaining to occupational injuries in the tea plantation sector in India, but the incidence of agricultural injuries in India has been found to be 3 per thousand per year.[6] In Sri Lanka, a total of 2391 accidents were reported from 161 estates employing 115,000 employees[7](an incidence of 20.79 injuries per 1000 or 2.08 per 100 workers per year), based on responses to a comprehensive questionnaire sent to the estates. In comparison, an incidence of 36% per year certainly warrants attention. However, there may be a possibility of over-reporting of accidents and injuries in this study as the survey was conducted by the administration of an interview schedule individually to the study population.

The nonfatal injury ratio for men to women was nearly 2:1, whereas the fatal injury ratio was about 11:1.[8] This study found that although a greater proportion of men had been injured at the workplace and the injury rate per 100 working days was also greater for male workers, there was no significant association between gender and injury.

Most of the workers had suffered an injury which was minor in nature (cuts, abrasions and contusions), requiring only first aid or minor treatment. This is explained by the fact that most workers (especially women workers) carry out their tasks in the field walking barefoot over a rough terrain and are prone to injuries with stones, thorns, etc. The Sri Lankan study also stated that the reasons for cuts and bruises were attributed to the improper use of sharp cutting devises such as knives and shears, and also the non use of protective footwear.[7]

No literature was found attributing multi-tasking to increased proneness to injury. Workers handling multiple tasks face an increased risk of getting injured probably due to several factors such as inadequate training or experience in one particular job, physical and psychological factors, compared to a worker performing the same task routinely. These hypotheses need to be investigated further.

This study demonstrates the mismatch between the categorization of risks as perceived by the workers versus the actual occurrence of work-related injuries – this is especially so since actual incidents remain longer in human memory. In this study, cuts and bruises were the most common injuries suffered by these workers in the course of the year, yet the risk rating assigned to cuts and bruises by the same workers was “very low”, pointing to the fact that even a very frequently occurring injury may not be perceived by workers as posing a significant risk. This in essence demonstrates the value of the Risk Rating Matrix which also takes into account the outcome of the injury, which may not be very severe.[9]

To conclude, continued monitoring of the risk assessment by the workers could help in a planned reduction of commonly occurring injuries by agreeing on a specified risk limit. For example, the safety policy of an estate could incorporate a statement that the perceived risk rating of, say, sprains and dislocations should at no point of time be allowed to exceed “very low”. The reasons why occupational injuries/ailments (in this case backache and insect bites) have been assigned a higher risk rating score should be looked into and corrective steps should be taken.

## References

[1] (2001). United Planters Association of Southern India. The Planters’ Chronicle.

[2] Muthiah S (1993). A Planting Century.

[3] Srinivasan S (2003). Srinivasan S. The Factories Act, 1948 with the Tamil Nadu Factories Rules 1950; the Tamil Nadu Factories (Welfare Officers) Rules 1953 and short notes of cases.

[4] V Subramaniam (2001). The Plantations Labour Act, 1951 with Tamil Nadu Plantation Labour Rules.

[5] National Program for Control and Treatment of Occupational Diseases, Burden of Occupational Diseases in Injuries. National Institute of Health and Family Welfare. http://www.nihfw.org/NDC/DocumentationServices/NationalHealthProgramme/NATIONALPROGRAMMEFORCONTROL.html.

[6] Leigh J, Macaskill P, Kuosma E, Mandryk J (1999). Global Burden of Disease and Injury due to Occupational Factors. Epidemiology.

[7] Asia-Pacific Regional Network on Occupational Safety and Health Information; ILO/EFC Plantation Safety and Health Monitoring Project 1997/Report produced by the Employees Federation of Ceylon, Colombo. http://wwwiloorg/public/english/region/asro/bangkok/asiaosh/country/srilanka/sloshteahtm.

[8] International Labour Office. World Day for Safety and Health at Work 2005: A Background Paper. http://www.ilo.org/public/english/bureau/inf/download/sh_background.pdf.

[9] Hazardous Substance Training record and register. Murdoch University. http://wwwoshmurdocheduau/proc-guide/Hazardous%20Substances%20Procedures%20Manualpdf.

